# The hologenome concept and its implications for animal breeding in the multi-omics Era

**DOI:** 10.1590/1678-4685-GMB-2025-0241

**Published:** 2026-05-25

**Authors:** Tainã Figueiredo Cardoso, Luciana Correia de Almeida Regitano

**Affiliations:** 1 Embrapa Pecuária Sudeste, São Carlos, SP, Brazil.

**Keywords:** Host-microbiome, microbiota, metagenome, metatranscriptome, metabolome

## Abstract

The hologenome is an evolutionary concept in which a host and its associated microorganisms together compose a single unit of selection, known as the holobiont. Bidirectional interactions between the host and the microbiota genomes shape complex phenotypes and have implications for evolution, adaptation, and breeding. Advances in multi-omics technologies now enable a comprehensive investigation of host-microbiota interactions, allowing their integration into breeding programs and revealing novel targets for enhancing animal health, productivity, and sustainability in agriculture.

## Introduction

Traditional biology, which defines an organism (*e.g*., animals or plants) as an isolated genetic entity, has been reshaped by the growing understanding of organisms’ interactions with associated microbial communities. The concept of the hologenome was first explicitly introduced by Richard Jefferson in 1994 and describes the host and all its symbiotic microorganisms as a single biological unit (Bordenstein and Theis, 2015). This genetic unit is characterized as a functional and evolutionary unit, comprising bidirectional interactions between the host and associated microbes that influence the variation of complex phenotypes (Nyholm et al., 2020). For example, the host genome can contribute to the formation of ecological niches for microorganisms and influence their gene expression, whereas microorganisms, in turn, can influence the expression of host-specific genes and produce systemic changes within the host organism.

This synergistic relationship between the host and its microbiota can generate new and emerging phenotypes that neither the host nor the microbes could produce independently (Haag, 2018). This relationship is a central principle of the hologenome theory and has direct implications for host evolution, ecology, and adaptation (Pietroni et al., 2025). In addition, symbiotic microorganisms can be transmitted vertically from parents to offspring through various mechanisms, ensuring the continuity of these relationships across generations. This vertical transmission, combined with horizontal transmission of microbes (e.g., from the environment or through social interaction), contributes to the establishment and maintenance of the holobiont (Roughgarden et al., 2018; Gurczynski et al., 2023).

Advances in microbiology, including genomics, metagenomics, transcriptomics, proteomics, and metabolomics, have enabled a comprehensive study of these complex interactions in both the host and microbiota. Understanding these interactions is essential for advances in areas such as agriculture, health, and conservation, where management of the hologenome can optimize animal performance and adaptation to environmental challenges (Nyholm et al., 2020). This review aims to demonstrate that integrating the hologenomic perspective not only enhances our understanding of evolution and adaptation but also opens up new opportunities and strategies for improving animal health, promoting productivity and sustainability of livestock systems.

## The hologenome concept in evolutionary biology

The hologenome concept expands traditional evolutionary theory by recognizing the dynamic, co-evolving, or cooperative relationship between macroorganisms and their microbiota, and the multiple ways these associations are inherited and can be shaped by natural selection (Roughgarden et al., 2018). However, despite the recognition of the importance of host-microbiota interactions, the concept of the holobiont as a unit of selection is still under debate. Some authors argue that natural selection can act on the host-microbiota assemblage as a whole, while others propose that holobionts should be seen as units of cooperation rather than as direct units of selection (Stencel and Wloch-Salamon, 2018; Almudi et al., 2024).

Importantly, both views in the debate (i.e., the holobiont as a unit of selection or as a unit of cooperation), may be valid depending on specific ecological and evolutionary contexts (Bordenstein and Theis, 2015). However, what really stands out in this debate is the dynamic nature of the interactions between the host and its microbiota. The continuous and adaptive host-microbiome interaction shapes the characteristics and fitness of the holobiont. Microbial symbionts can contribute to host adaptation by providing essential functions such as nutrient acquisition, stress tolerance, and immune modulation (Haag, 2018), highlighting the complexity and importance of host-microbiota relationships.

## Transmission of the microbiota: vertical inheritance, horizontal dissemination, and genetic exchange

**Figure 1 -:**
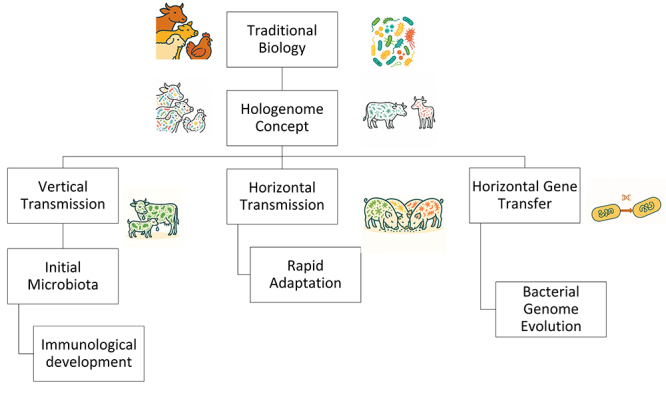
Vertical and horizontal routes of microbiota transmission and genetic exchange in livestock systems.

The mode of microbiota transmission can profoundly influence microbial ecology and host adaptation across animal species (Figure 1). For instance, newborn worker bees receive a microbiota inoculum from healthy nurse bees (Mueller and Linksvayer, 2022). Newborns primarily acquire their initial gut microbiota vertically from their mothers during childbirth and breastfeeding, as well as through vaginal secretions and milk, influencing the establishment of the neonatal microbiota and its immunological development in different species such as cattle (Moeller et al., 2018), swine (Zhang et al., 2024 a ), and chickens (Shterzer et al., 2023). Horizontal transmission occurs through contact with family members, surroundings, or diet, enabling a rapid response to environmental changes and the spread of new traits. Zhang *et al*. (2022a) showed that social interaction facilitates the horizontal transmission of the microbiota both intra- and interspecifically in co-housing goats and pigs. They showed that the transmission of microbial strains within the same species (intraspecific transmission) was the main pattern, accounting for 82.25% of transmitted strains, followed by transmission between species (interspecific transmission) at 5.19%. Similarly, cattle and horses sharing the same accommodation shared major microbiota phyla, including *Proteobacteria*, *Firmicutes*, *Bacteroidetes,* and *Actinobacteria* (Park and Kim, 2020).

In addition, some studies have demonstrated horizontal gene transfer in microbiota (particularly resistance genes) in farm animals. Horizontal gene transfer (HGT) is a key driver of the evolution of bacterial genomes, allowing bacteria to rapidly acquire new functions and adapt to changing environmental conditions (Schwarzerova et al., 2023). 

Schwarzerova et al. (2023) described horizontal gene transfer in chicken and pig gut microbiota. They analyzed the genomes of 452 gut microbiota isolates from chickens and pigs and identified more than 6,000 suspected HGT genes. The main reservoirs were *Phocaeicola* spp. (*Bacteroidaceae*) and *UBA9475* spp. (*Oscillospiraceae*). In addition, this study highlighted that different species and strains of the same genus typically carry different sets of mobilized genes, with an enrichment of genes associated with intracellular trafficking, and secretion, and with DNA repair.

Qi et al. (2023) analyzed the distribution of β-lactamase resistance genes on dairy farms. They found that over 98% of blaTEM genes (genes conferring resistance to β-lactam antibiotics) were present in meal, water, and milk samples. In addition, they identified that mobile genetic elements (*tnpA-04* and *tnpA-03*) facilitated the transfer of these genes between environmental compartments. This capacity for horizontal gene transfer, including antibiotic resistance genes, adds a layer of complexity and dynamism to the holobiont context, especially in animal production environments.

## Host-microbiota interactions shaping animal phenotypes

Host-microbiota interactions are fundamental drivers of animal phenotypes, influencing immunity, metabolism, endocrine and neuromodulation, and, consequently, productive traits. The microbiota itself plays a crucial role in digesting complex feed components, supporting growth, and conferring disease resistance. Table 1 summarizes key findings demonstrating this interaction in complex phenotypes.

**Table 1 -:** Summary of multi-omics studies investigating host-microbiome interactions and their effects on phenotypic traits across livestock species.

Reference	Species	Phenotype Type	Type of Omics Used	Main Results
Martínez-Álvaro et al. (2022)	Cattle	Methane emission	Host genotype, metagenomic	Host genomic significantly influences microbiome variation, with heritabilities ranging from 0.13 to 0.61. This control is evident in the strong genetic correlation found between methane emissions and 29 microbial genera, 22 RUGs, and 115 microbial genes.
Malheiros et al. (2023)	Cattle	Feed efficiency	Metabolomic	Dimethylamine, Aspartate, Fatty acid C14:0 and hypoxanthine were significantly associated with RFI
Wang et al. (2023)	Goat	Growth Rate	Metataxonomy, host serum parameters and volatile fatty acids	A cluster comprising Succinivibrio and various Prevotellaceae members was identified playing a role in promoting gut fermentation, host metabolism (including amino acid biosynthesis and starch and sucrose metabolism), and enhancing host growth rates.
Mamun et al. (2020)	Sheep	Susceptibility/Resistance to parasite	Automated ribosomal intergenic spacer analysis (ARISA) and Metataxonomy	A substantial parasite burden was linked to significant alterations in community composition, including changes in the relative abundances of Firmicutes and Bacteroidetes
Nuñez et al. (2025)	Swine	Feed efficiency	Metataxonomy	Microbial heritability estimates were found to be 0.44 ± 0.13. The ASVs significantly influencing both FCR and RFI were identified within the genera *Lactobacillus*, *Limosilactobacillus*, NK4A214 group, *Lachnospiraceae UCG-001*, and *Eubacterium nodatum group*.
Guitart-Matas et al. (2025)	Swine	Antimicrobial resistance	Metagenomics and metatranscriptomics	Gentamicin rapidly inhibited bacterial protein synthesis, as evidenced by altered expression of ribosomal-related genes.
Fonseca et al. (2024)	Poultry	Growth performance	Metataxonomy	A probiotic significantly improved feed conversion ratio and growth. Feed supplements did not affect alpha diversity but did impact microbial beta diversity.
Johnson et al. (2018)	Broiler Chicken	Performance traits	Metataxonomy	Several bacterial taxa were highly positively correlated with performance in young birds or consistently correlated with performance over time, i.e, *Bacteroides*, *Butyricimonas*, *Faecalibacterium*, *Parabacteroides*, and *Sutterella* in the gut.
Zhang et al. (2020)	Nile tilapia	Energy harvest	Metataxonomy	*Citrobacter* spp., modulated intestinal microbial composition and increased high-fat diet-induced lipid accumulation in mesenteric adipose tissue and increased intestinal permeability in the host

## Influence on productive traits and feed efficiency

The microbiota’s role in digestion provides essential nutrients and energy. In ruminants, for instance, approximately 70-80% of energy needs are met by volatile fatty acids (VFAs), primarily acetate, propionate, and butyrate, which originate from microbial fermentative processes in the rumen (Abecia et al., 2018). The composition of the rumen and fecal microbiome, as well as the microbial functions, are associated with feed efficiency in cattle. Holstein cows with better feed efficiency showed a higher abundance of *Bacteroidetes* and *Prevotella,* and a lower abundance of *Methanobacteria* and *Methanobrevibacter* (Delgado et al., 2019). 

In Nelore cattle, Conteville et al. (2023) found that the ruminal abundance of *Anaeromyxobacter*, *Actinomadura*, *Sorangium*, and *Rubrobacter* genera was associated with a decrease in the residual feed intake (RFI) values (more efficient cattle), while in the fecal microbiome, *Succinivibrio* and *Marinomonas* were identified as significantly associated with efficient cattle. In addition, these authors found rumen taxa associated with feed efficiency in cattle were characterized by a significantly higher abundance of functions linked to the carbohydrates metabolism, such as mono-, di-, and oligosaccharides, and amino acids. 

Zhou et al. (2025) identified amplicon sequence variants (ASVs) linked to porcine growth traits. They found that the gut microbiota can explain approximately 2.17-3.00% of the phenotypic variance in growth traits in pigs. *Prevotella* and *Oscillospiraceae* ASVs were negatively correlated with pig growth, while *Muribaculaceae* and *Rikenellaceae* ASVs showed positive correlations in fecal microbiota samples. In addition, Hu et al. (2024) showed that three core-predominant gut microbes (including *P. succinatutens*, *P. copri*, and *O. valericigenes*) isolated from pigs were capable of inducing morpho-physiological changes in germ-free mice, *e.g.*, increasing organ indices (heart, spleen, and thymus), decreasing gastrointestinal length, and affecting several metabolic pathways.

In poultry, gut microbiota impact immunity, nutrient digestion, and feed efficiency (Kogut, 2013). The use of antibiotic growth promoters increases growth and feed efficiency, probably by reducing the bioenergetic costs associated with intestinal inflammation. In addition, while host genetics contribute to approximately 39% of residual feed intake (RFI) variation, gut microbiota, particularly cecal microbiota, can account for as much as 28% of the variation in RFI in chickens (Wen et al., 2021). Low abundances of *Akkermansia muciniphila* in the duodenum and *Parabacteroides* in the cecum, as well as high abundances of cecal *Lactobacillus*, *Corynebacterium*, *Coprobacillus*, and *Slackia* were associated with better feed efficiency (Wen *et al*., 2021).

Beyond taxonomic associations, microbial metabolites provide a functional link between gut microbiota composition and host phenotypes. In a study in dairy cows, Sun et al. (2015) showed that microbial metabolites involved in glycine, serine, and threonine; tyrosine; and phenylalanine metabolisms were associated with milk yield and milk protein quality, acting as phenotypic biomarkers of dairy efficiency via biofluid integration. Recently, Ghaffari et al. (2025) demonstrated a complex interaction among dietary factors, liver and gastrointestinal functions, and the gut microbiome in shaping the fecal metabolite profile of dairy cows. Similar results were described by Malheiros et al. (2023), who showed that different nutritional interventions can alter ruminal and fecal metabolites and influence feed efficiency and water intake traits in Nelore bulls. They identified that aspartate and dimethylamine in ruminal fluid were negatively associated with RFI, whereas acetone, aspartate, choline, and dimethylamine compounds were negatively associated with water intake. Additionally, fecal metabolites C14:0 and hypoxanthine were significantly associated with RFI, while methylamine was positively associated with feed efficiency. These findings indicate that metabolomic profiles capture downstream functional effects of microbiota composition, bridging microbial ecology and host performance traits.

Although relatively understudied, the virome can modulate host immune responses and gene expression, as well as microbiome structure, affecting trans-kingdom dynamics via phage-host regulation (Yan and Yu, 2024). In Holstein dairy cows, Liu et al. (2025) showed differences in the composition of viral operational taxonomic units, lytic/temperate cycles, and viral metabolic genes (AMGs) associated with feed efficiency. They suggested lytic viruses could lyse host-beneficial bacteria linked to favorable cattle phenotypes and/or AMGs could modulate host metabolism as possible mechanisms for feed efficiency modulation by rumen viruses. In beef cattle, ruminal virome profiles were associated with carcass weight and marbling, and different viral genes, mainly those categorized as glycosyl hydrolases, were identified, suggesting a possible role in the conversion of fiber into energy (Sato, 2025). In addition, in swine and poultry, the gut viromes shift developmentally, and viral genes, including CAZymes and carbohydrate-active enzymes can modulate bacterial communities linked to growth and feed conversion (Chen et al., 2023; Yang et al., 2026).

## Impact on immunity and disease resistance

The microbiome has a substantial impact on the host’s systemic immunity and can influence many aspects of health and adaptive immune function (Broom and Kogut, 2018). Germ-free chickens have a less developed and mature intestinal mucosa and a significant reduction in cecal tonsil lymphoid tissue (Gaboriaud et al., 2021). In addition, Saint-Martin et al. (2024) demonstrated that the intestinal microbiota can modulate mucosal immunity in the lungs during infection with avian influenza viruses. This study also showed the central role of the microbiota and its metabolites in the homeostasis and immunity, since butyrate, produced by bacteria, exhibited an antiviral role in chicken respiratory epithelial cells, regulating the interferon-stimulated genes, such as the *OASL*, via the transcription factor Sp1.

The microbiota can also confer greater resistance or susceptibility to diseases. Zhao et al. (2022 a ) showed that Min pigs had stronger resistance to acute colitis compared to Yorkshire pigs. They found some pathogenic microorganisms (*i*.*e*., *Desulfovibrio*, *Bacteroides*, and *Streptococcus*) enriched in sick animals of both breeds, while beneficial microorganisms (*Lactobacillus*, *Clostridia*, *Eubacterium*, *Ruminococcaceae*, and *Christensenellaceae*) were enriched in diseased Min pigs and were associated with the protection of the intestinal barrier. In sheep, Yan et al. (2024) showed that microorganisms such as *Lachnospiraceae*, *Oscillospiraceae* and *Clostridiaceae* were positively associated with resistance to DSS-induced inflammatory enteritis, while *Bacteroidaceae* and *Treponemataceae* were positively associated with susceptibility.

Prophages in intestinal bacteria encode antimicrobial resistance factors, altering microbial composition and, indirectly, the mucosal barrier against invaders, thus influencing immunity and disease resistance (Chen et al., 2025). In pigs, He et al. (2021) showed that enteric viromes with persistent parvoviruses were able to modulate secretory IgA, conferring resistance to viral/bacterial enteritis during weaning. In poultry, post-hatch intestinal phages train macrophages against Salmonella, impacting survival in commercial flocks (Yang et al., 2026). In addition, some studies suggest that vertical transmission diversifies intestinal viruses, impacting health and biosecurity phenotypes in production systems (Dow et al., 2015; He *et al*., 2021; Chen *et al*., 2025).

## Metabolism regulation

The intestinal microbiota is an integral part of multiple physiological processes in the host. Dysbiosis can alter the ratio between beneficial and harmful intestinal bacterial species, affecting the host’s ability to harvest energy from food and respond to energy intake (Kang et al., 2022), as well as increasing the levels of circulating bacterial products that promote inflammation. In cattle, gut or ruminal dysbiosis contributes to inflammation and can lead to various diseases, including mastitis and metabolic disorders such as rumen acidosis (Elmhadi et al., 2022; Zhao et al., 2022 b ). In pigs, lung inflammation induced by *Mycoplasma hyorhinis* infection was associated with gut microbiota dysbiosis and intestinal barrier dysfunction (Zhang et al., 2022 b ). This dysfunction results in intestinal hyperpermeability and increased translocation of bacterial endotoxins, such as lipopolysaccharides (LPS), to the host circulation culminating in a dysregulation of intermediary metabolism, reduced feed intake, and both local and systemic inflammation (Ringseis and Eder, 2022). 

## Neural development and behavioral traits

The gut-microbiota-brain axis involves multiple biological processes that mediate communication between the gut microbiota and the brain, including, but not limited to metabolites, neurotransmitters, immune responses, and cellular signaling molecules that modulate both gastrointestinal and brain functions (Cryan et al., 2019). 

Recent studies in mice comparing germ-free and conventionally colonized lines have shown that maternal microbiota influences the uterine-dependent development of the hypothalamic paraventricular nucleus, a central endocrine and behavioral regulator (Milligan et al., 2025). Neurogenesis and myelination were also shown to be impaired in germ-free mice, with the latter also being affected by postnatal antibiotic therapy, while several neurodevelopmental diseases have been associated with dysfunctions of the gut-microbiota-brain axis (Morais et al., 2021). Gut-derived CD4^+^ T-cells have been identified in the subfornical organ of the human and mice brain, and are involved in maintaining the homeostasis of the gut-brain axis (Yoshida et al., 2025). 

Microbial metabolites appear to be key mediators of microbiota-directed effects on animal behavior, as highlighted by a recent review by Ntiri and Chun Nin Wong (2025). Metabolites such as SCFAs (i.e., acetate, propionate, butyrate), secondary bile acids, microbial volatiles, and indoles can modulate at least seven behaviors: nutrition/foraging, olfaction, circadian rhythms, reproduction, locomotion, aggression, and social interactions (Ntiri and Chun Nin Wong, 2025). These compounds interact with host receptors (e.g., FFARs for SCFAs, FXR/TGR5 for bile acids), influencing neurotransmitter release (GABA, serotonin), synaptic and epigenetic plasticity, generating dose-dependent behavioral variation. In pigs, SCFAs and tryptophan derivatives regulate the intestinal barrier, stress responses via the hypothalamic-pituitary-adrenal axis, and behaviors such as tail biting via cortisol reduction and serotonin increase (Rabhi et al., 2020; Verbeek et al., 2021).

Conversely, the host nervous system is also capable of modulating the gut microbiota composition (Toledo et al., 2025). Regarding farm animals, differences in ruminal microbiota were described between individuals with contrasting temperament score in a Chinese sheep breed (Wu et al., 2023), while in *Bos indicus* cattle, a total of seven bacteria genera (including *Clostridium sensu stricto* 1, *Turicibacter*, *Cellulosilyticum*, *Christensenellaceae R-7 group*, *UCG-010*, *Oscillospiraceae*, and *Rikenellaceae RC9 gut group*) were associated with reactivity to handling (da Silva *et al*., unpublished data). 

## Technological advances enabling hologenomic research

Hologenomic analysis integrates molecular data from the host and its microbiota, using multiple layers of information to investigate complex interactions and biological functions. The main types of omics approaches employed to this end include genomics/metagenomics (DNA), transcriptomics/metatranscriptomics (RNA), proteomics/metaproteomics (proteins), metabolomics (metabolites), and epigenomics (regulatory modifications), which together provide different layers of biological information (Nyholm et al., 2020). An overview of these omics and their applications in hologenome studies is presented in Table 2.

**Table 2 -:** Overview of omics approaches and their applications in hologenome studies.

Omics Type	Generated Information	Main Applications
Genomics/Metagenomics	Sequence and variation of host/microbiota genes	Genetic diversity, functional potential, species identification, evolution
Epigenomics	Chemical modifications in chromatin (e.g., methylation, histone modification)	Gene expression regulation, phenotypic plasticity
Transcriptomics/Metatranscriptomics	Gene expression (RNA) of the host/microbiota	Response to stimuli, gene regulation, adaptation
Proteomics/Metaproteomics	Protein profile	Metabolic functions, signaling, protein-protein interactions
Metabolomics/Metametabolomics	Produced metabolites and their quantities	Metabolism, biomarkers, health/disease
Lipidomics	Lipid profile	Membranes, signaling, energy metabolism

**Figure 2 -:**
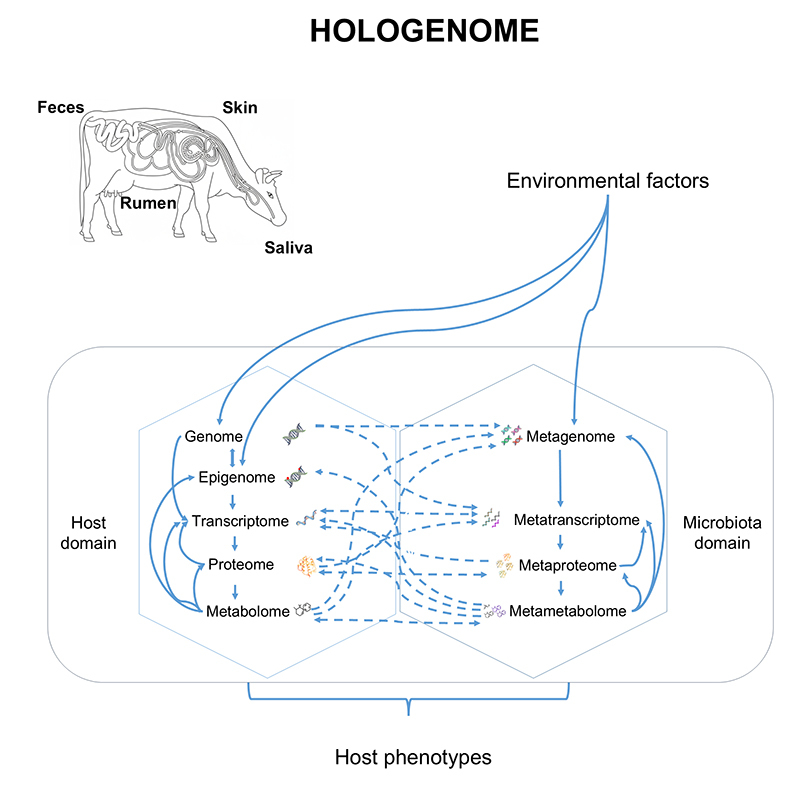
Conceptual representation of the hologenome framework integrating host and microbiota multi-omics interactions. The figure illustrates the bidirectional interactions between the host domain and the microbiota domain, influenced by environmental factors. These multilevel molecular interactions jointly shape host phenotypes, such as feed efficiency, methane emission, and disease resistance. Sampling sites such as rumen, feces, saliva, mucous, and skin are key sources for host-microbiome multi-omics analyses. Solid arrows represent hierarchical molecular cascades within each domain, while dashed arrows indicate cross-domain interactions between host and microbiota omics layers, reflecting molecular crosstalk and co-regulation.

**Figure 3 -:**
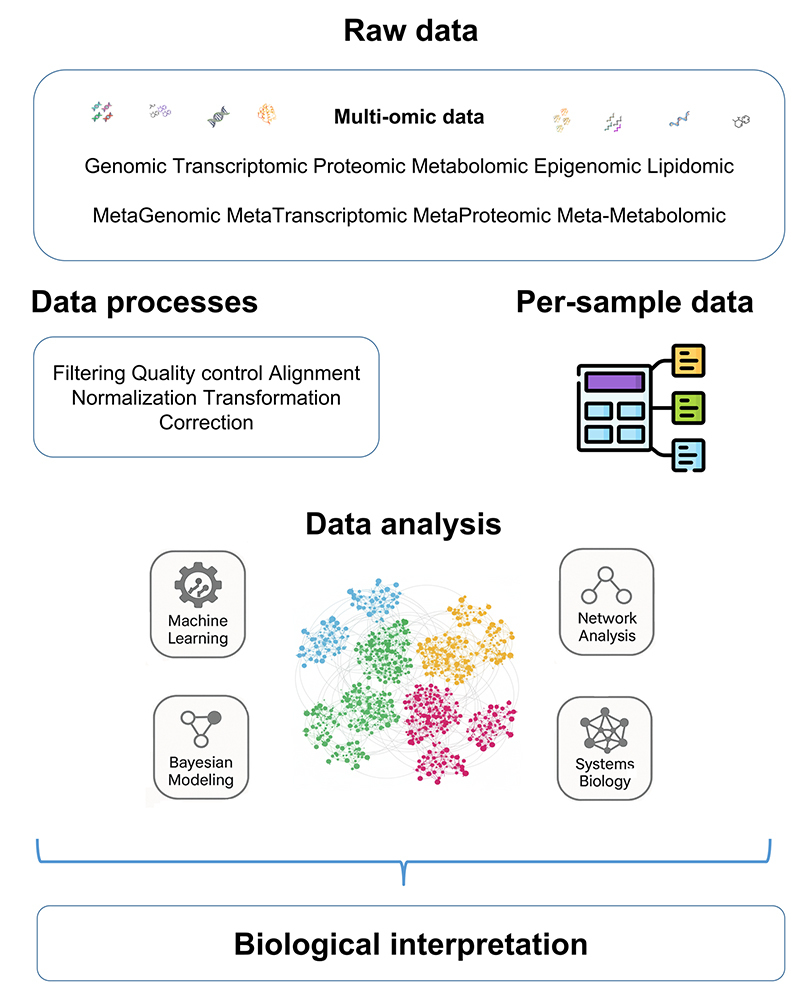
The integration of different omic layers of the host and its microbiota. Information from multiple omic layers should be processed respecting the characteristics of each data set, allowing for joint adjustments per sample, such as integrative batch corrections. Measurements per sample can be modeled together by using different approaches and post-hoc integrations are performed for biological inferences.

The integration of the omic domains of the host and the microbiota helps elucidate how external factors, such as environmental factors, can modulate these interactions at different levels. The hologenomic approach also allows for a systemic understanding of how holo-omic data (from both the host and the microbiota) interact and directly influence phenotypes of productive interest, such as growth rate and disease resistance (Figure 2). The findings pave the way for innovative applications in health, agriculture, and ecology. However, the integration of this complex data remains highly challenging (Figure 3). The main analytical challenges and integration strategies commonly employed in hologenomic research are summarized in Box 1.

## Implications for animal breeding

### Evidence of microbiome heritability

Among the many factors that shape microbiota composition, recent scientific data suggest a significant role for host genetic variation across a wide variety of plant and animal hosts. There are three main approaches to investigate this role: estimating microbial heritability, analyzing the relationship between microbiota taxon abundance and specific host candidate genes, and identifying genome-wide host variants associated with microbiota taxon abundance using GWAS (Ryu and Davenport, 2022). 

Heritability is a statistical concept that quantifies the proportion of phenotypic variation that can be attributed to genetic variation, ranging from 0 to 1. It is worth noting that heritability is related to allele frequencies; thus, it is a population parameter and cannot be assumed to represent a general condition for all populations. Heritability can be defined in a stricter sense, in which only the additive value intrinsic to a given allele is considered; thus, it quantifies the proportion of phenotypic variance explained by additive genetic variance. This stricter concept of heritability is usually applied to animal breeding, as only alleles, not genotypic combinations, are inherited. 

Accurately estimating heritabilities of microbiome components is challenging because these estimates often rely on relatives, who frequently share diets, behaviors, and environments (Grieneisen et al., 2021). For instance, in the particular case of mammals, full siblings and maternal half-sibs share one of the primary sources of microbiota inoculum. An additional challenge arises from the compositional nature of microbiome data. Generally, DNA-based microbiota profiling studies rely on the relative abundance of specific taxonomic groups compared to the total abundance of all taxa. In this case, biases can emerge from co-abundance, particularly affecting the most abundant taxa (Bruijning et al., 2023).

Nonetheless, the available data clearly show that the heritability varies among taxa, ages, and environments. For example, in humans, large studies on twins found the bacterial family *Christensenellaceae* to be the most heritable, while taxa from the *Bacteroidetes* phylum were not heritable (Goodrich et al., 2016). In baboons, a 14-year dataset found 97% of the microbiota as significantly heritable, typically with low heritability estimates, which tended to increase with age and with reduced diet diversity (Grieneisen et al., 2021). In pigs, GWAS identified host genomic regions associated with the relative abundance of multiple bacterial taxa, supporting the idea that the microbiome itself can be considered a complex of correlated features. Ramayo-Caldas et al. (2021) performed microbial-wide association studies for 21 immunity traits and the relative abundance of gut bacterial communities in pigs. Results suggested a polymicrobial nature of the immunocompetence in pigs and revealed associations between pigs’ gut microbiota composition and 15 of the analyzed traits. The strongest association was observed between the relative abundance of Chlamydia, Peptococcus, and Streptococcus with the profile of lymphocytes phagocytosis. In addition, considering the joint effects of host-genome and gut microbiota, these two sources of variation explained from 29.9% to 51.7% phenotypic variance of the analyzed immunity and hematological traits. These findings provide a quantitative genetics basis for incorporating microbiome information into animal breeding frameworks.

### Indirect selection of microbiota features

Changes in microbiome composition induced by animal domestication include, for instance, the selection of taxa associated with higher feed efficiency and the horizontal transmission of typical human taxa to domesticated animals. Correlated responses in gut microbiota composition following artificial selection for host traits are expected, given their effects on phenotypes and their heritability. For instance, long term divergent selection for 56-day body weight in chickens affected not only the composition of the microbiota, but also the correlations among taxa and their heritabilities (Meng et al., 2014). Changes in microbiome composition were also observed between two rabbit lines selected for RFI and average daily gain, and their original population (Drouilhet et al., 2016). In rabbits, changes at the genus level were observed in the gut microbiota between two artificially selected divergent lines for intramuscular fat deposition (Martínez-Álvaro et al., 2024 b ). In pigs, Camarinha-Silva et al. (2017) showed that eight from 49 investigated bacteria genera showed a significant host heritability (ranging from 0.32 to 0.57), and showed that the fraction of phenotypic variance explained by the microbial variance was 0.28, 0.21, and 0.16 for daily gain, feed conversion, and feed intake, respectively, highlighting the potential for indirect selection responses mediated by the microbiome. 

### Microbiome as an intermediate phenotype

Indirect selection for correlated traits based on microbiota signatures associated with host traits is also possible, although it remains overlooked. Nonetheless, the response to artificial selection for two enterotypes present in the population of 60 days-old pigs resulted in two lines enriched for each enterotype. This microbiota-based selection impacted the growth rate during the post-weaning period (Larzul et al., 2024). Enterotypes, *i.e.,* specific combinations of microorganisms within the microbiota of a population (Costea et al., 2018), are a useful approach for capturing the compositional effect (signatures) of the microbiota on traits. In dairy goats, enterotypes were associated with differences in milk yield, fat-corrected milk yield, and milk fat yield. Additionally, differences were found for concentrations of total volatile fatty acids (VFA) in the milk, serum glucose, and total bile acids levels (Wang et al., 2025). 

In broiler chickens, Díaz-Sánchez et al. (2019) found significant differences in microbiota composition between phenotypically distinct lineages. Using a random forest classification model based on microbiota composition, the authors were able to accurately classify the animals into high and low feed conversion groups but were only successful in classifying the animals for weight gain in one of the two lines. Moreover, distinct microbiota features were identified as the best predictors in each line. In rabbits, only four gut microorganisms were enough to assign an animal to its divergent intramuscular fat line (Martínez-Álvaro et al., 2024b). 

Structural equation models (SEM), as introduced in livestock quantitative genetics by Gianola and Sorensen (2004) have been adapted to explicitly model direct and indirect effects, allowing the decomposition of host genetic effects into components mediated by the microbiota and components acting directly on the phenotype (Haas et al., 2023). These authors demonstrated in chickens that microbial diversity (Jʹ) mediates genetic effects on feed efficiency via SEM-GWAS, quantifying indirect effects of SNPs (up to 10-15% total variance). 

In addition, bidirectional Mendelian randomization (MR) was also applied (Teng et al., 2024; Xue et al., 2024; Zhang et al., 2024 c ). By using MR method, Xue *et al*. (2024) found a relationship between *Selenomonas bovis* and rumen carbohydrate metabolism, mediated by bacterial chemotaxis and a two-component regulatory system, impacting feed utilization efficiency of dairy cows. This approach was used by Zhang *et al*. (2024c) in sheep with genotype data from whole-genome resequencing, 16S rRNA sequencing and multilevel fat deposition-traits data. They found eight causal associations between microbial genera, including *Butyrivibrio*, *Olsenella*, *p-2534-18B5 gut group*, *Prevotellaceae UCG-003*, *Flexilinea*, *Suttonella*, and *Pseudobutyrivibrio*, and fat deposition-traits. This explicit decomposition of direct and microbiota-mediated genetic effects provides the conceptual basis for hologenomic selection, in which selection decisions can jointly exploit host genomic information and the heritable fraction of microbiome features.

### Inclusion of microbiota in genomic selection

Recent reviews have highlighted the microbiome as an important source of phenotypic variation in animal breeding and discussed the statistical and conceptual challenges associated with its integration into selection programs (Maltecca et al., 2020; Peixoto et al, 2021; Venegas et al., 2026).

The term *microbiability* has been proposed by Difford et al. (2018) to describe the proportion of phenotypic variance explained by the microbiota. To estimate the microbiability, a covariance matrix is used to summarize the microbial resemblance between individuals. The method used to build this relationship matrix remains under debate, as accuracy is sensitive to the method employed. In 2019, Maltecca et al. (2019) demonstrated that the microbiome composition can be effectively used as a predictor of growth and composition traits, particularly for fatness traits. Accuracy increased substantially due to the inclusion of microbiome information, with values ranging from approximately 0.30 for loin characteristics to over 0.50 for backfat in pigs. He et al. (2022) showed that the microbiability estimates for four growth and body composition traits ranged from 0.02 to 0.53, varying according to pig breed and method.

As data on microbiota composition accumulate at the population level for livestock, the inclusion of this information in predictive models has been increasingly proposed. This integration should be of particular interest for specific phenotypes, such as greenhouse gas emission-related traits and feed efficiency, which are expensive and laborious to measure. In a large study involving 1,200 ewes and 3,139 rumen samples profiled by restriction enzyme reduced representation sequencing (RE-RRS) of the metagenome, Hess et al. (2023) reported higher microbiability estimates for methane-related traits than heritability itself. Accordingly, in this study, models considering metagenomic relationships explained more variation than those including only genomic relationships, and accuracy improved when both metagenomic and host genomics relationships were considered. In cattle, Roehe et al. (2016) showed that host genetics and rumen microbiome composition jointly influence methane emissions and feed efficiency. They found a set of microbial genes that explained 81% and 86% of the methane emissions and feed conversion efficiency variation and were clustered in distinct functional gene networks. Some methanogenesis-related genes (e.g. *mcrA* and *fmdB*) were associated with methane emissions, while host-microbiome cross talk genes (e.g. *TSTA3* and *FucI*) were associated with feed conversion efficiency. That study provided early evidence that microbial features can serve as informative predictors in breeding programs targeting both productivity and environmental sustainability.

Camarinha-Silva et al. (2017) extended classical BLUP methodology by incorporating microbial features as random effects. Within this framework, genomic best linear unbiased prediction (G-BLUP) and microbial best linear unbiased prediction (M-BLUP) methods were applied, employing SNP data and microbiota composition, respectively, to predict the complex traits. M-BLUP showed higher prediction accuracies compared to G-BLUP across production traits. Specifically, M-BLUP accuracies were 0.41 for daily gain, 0.33 for feed conversion, and 0.33 for feed intake. In contrast, G-BLUP accuracies for the same traits were lower: 0.35, 0.23, and 0.20, respectively. The M-BLUP approach has since been adopted due to its flexibility, compatibility with established animal breeding pipelines, and ability to accommodate both single-feature and multi-feature microbiome-wide association analyses, as further demonstrated by subsequent developments in MWAS methodologies (Vollmar et al., 2020; Ramayo-Caldas et al., 2021; Haas et al., 2022). 

As with the primordial times of genomic selection, one of the limitations in integrating metagenome information into genomic selection is the number of individuals with both metagenomic, genomic and phenotypic data. A recent study combining the use of portable accumulation chambers to measure methane emission and RE-RRS and metagenome profiling demonstrated the predictive power of rumen metagenome information based on 4,585 mixed-sex lambs from multiple flocks and years, resembling the data structure of a breeding program (Bilton et al., 2025). 

### Hologenomic selection: integrating host genetics and microbiota into breeding decisions

**Figure 4 -:**
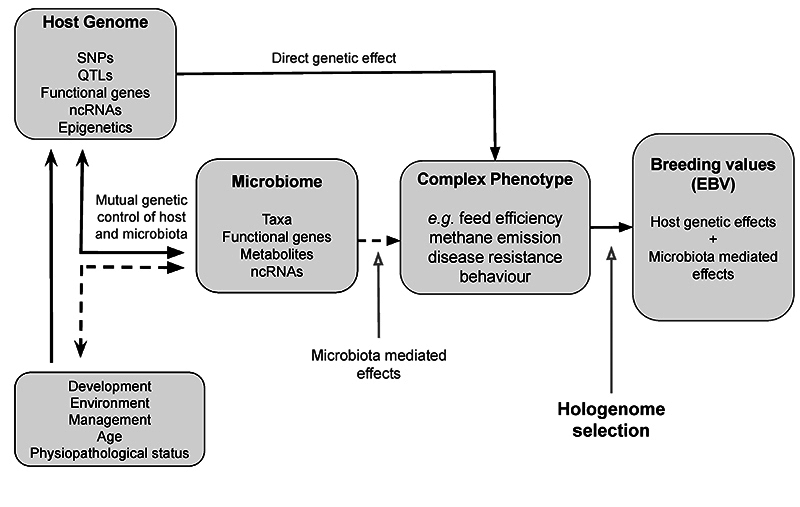
Conceptual framework of hologenomic selection in animal breeding. Host genomic variation affects complex phenotypes both directly and indirectly through its influence on the gut microbiome, which acts as an intermediate and partially heritable phenotype. Microbiota composition and function (including microbial taxa, genes, and metabolites) mediate part of the genetic effects on economically important traits. Hologenomic selection integrates host genomic information and the heritable component of microbiome variation, allowing breeding values to be decomposed into direct host genetic effects and microbiota-mediated effects, extending classical genomic selection.

While microbiability and M-BLUP approaches primarily focus on improving prediction accuracy, they do not explicitly define how microbiota-mediated effects should be exploited in selection decisions, motivating the development of hologenomic selection frameworks. In the hologenome context, the classical genomic selection can be extended, since instead of using only host SNPs, it would also integrate microbiota data into predictive models. The conceptual framework of hologenomic selection can be found in Figure 4.

In pigs, Weishaar et al. (2020) performed the first practical application of the hologenome selection, by proposing to divide the genetic value into two main components: i) direct (animal metabolic pathways) and ii) microbiota-mediated (effect of heritable microbial profiles), and using an index that combines both to improve feed efficiency and daily gain. In this study, they demonstrated that microbiota explains ~25% additional phenotypic variance in feed efficiency, justifying routine collections in breeding centers to accelerate genetic gains. This approach acknowledges that selection on host genotypes can indirectly drive favorable microbiome configurations, while also allowing the microbiome itself to contribute predictive information for selection. In 2021, Christensen et al. (2021) formalized mathematics of omics mediation in estimated breeding values, proposing a general framework for mixed multi-trait models incorporating inclusion of omics data (i.e., transcriptomics, metabolomics) and similar intermediate data (e.g., metagenome) in genetic evaluation between genotype and phenotype. 

Recently, by analysing longitudinal body weights during the finishing period, along with genomic and metagenomic data from beef cattle, Martínez-Álvaro et al. (2024a)  showed that combining microbial genes expression with average daily gain (ADG) increased prediction accuracy of genomic estimated breeding values by 11 to 22% relative to the direct breeding strategy (using ADG-traits only), whereas using only microbiome information achieved lower accuracies (from 7 to 41%). In pigs, adding microbiota information to the model considerably increased the prediction accuracies for the digestive efficiency traits (Déru et al., 2024).

### Complementary strategies for modulating the microbiota: management, nutrition, biotechnology

Besides host genetics, there are other factors that may influence microbiota composition, which can be strategically used to improve animal health and welfare. Management practices are fundamental in many aspects, including the levels of sanitary conditions arising from population density, manure accumulation, vaccination, deworming and use of antibiotics. Additionally, stress levels imposed by management practices, such as caging, castration, weaning, thermal comfort, and housing conditions are also crucial. These factors directly impact the source and composition of inoculum, the colonization capability of the microbiota, and the host’s immune defense.

Diet is also a key driver of microbiota composition, and changes in its formulation induce a cascade of metabolic changes (Palmonari et al., 2024). For instance, metatranscriptomic analyses revealed more pronounced functional and metabolic changes in the ruminal microbiome of *Bos indicus* than taxonomic changes (da Silva et al, 2025). Some specific dietary compounds (prebiotics), such as polysaccharides (Zhao et al., 2025), polyphenols (Bešlo et al., 2023), minerals (Juste Contin Gomes et al., 2021), and fat (Wu et al., 2024) may be used to modulate composition and function of the microbiota.

The use of probiotics (*i.e.* active microorganisms) and postbiotics (*i.e.* inactive microorganism or their constituents) in animal production has been proposed to replace antibiotics, based on their antagonism toward pathogenic microorganisms, or in the introduction of a beneficial microbiota that supports health and production traits (da Silva et al., 2024; Zhang et al., 2024 b ; Yue et al., 2025). Microbiota transplantation has also been proposed (Rahman et al., 2025), and it is well established in humans as a complementary treatment for specific diseases (Sahle et al., 2024). Given the complex nature of host genetics and other environmental factors, these latter strategies rely on continuous intervention.

### Challenges and future perspectives

Advances in hologenomic research have revealed the complex network of interactions between hosts and their microorganisms. However, significant challenges remain in consolidating this field and its practical applications in animal production. One of the main issues is the distinction between causality and correlation. Although several associations between microbiota and phenotypes have already been identified, establishing causal relationships requires rigorous experimental approaches and statistical models capable of dealing with the multivariate and temporal nature of the data.

Another critical point is the stability, transmissibility, and modularity of the microbiota. The microbiota is a dynamic ecosystem, highly sensitive to environmental, dietary, and genetic variations. The long-term stability and the capacity for both vertical and horizontal transmission of microorganisms are still poorly understood, which limits the likelihood of the effects of interventions. Functional modularity, i.e., the idea that specific microbial groups can act in a coordinated manner to influence certain phenotypes, is a promising approach but still requires experimental validation.

Finally, ethical, regulatory, and biosafety issues are gaining prominence as strategies such as microbiota transplantation, the use of genetically modified probiotics, and targeted manipulation of microbial communities evolve. A clean regulatory framework is needed to ensure the safety of these practices, considering ecological risks and potential side effects.

Thus, the future of hologenomic biology depends on effective integration between experimental approaches, computational modeling, ethical and policy frameworks. Continued interdisciplinary collaboration will be important to translate hologenomic knowledge into sustainable applications in animal production.

## Supplementary material

The following online material is available for this article:

Box 1 - Analytical challenges and integration strategies in hologenomic research.

## Data Availability

No new data was created in this work.
