# Supplementary material for: The hologenome concept and its implications for animal breeding in the multi-omics Era
Source: Genet Mol Biol. 2026 May 25;49(Suppl 1):e20250241. doi: 10.1590/1678-4685-GMB-2025-0241 (PMC13227325; doi:10.1590/1678-4685-GMB-2025-0241)
Supplement: Box 1 - [file 1415-4757-GMB-49-s1-e20250241-s1.pdf]

Genetics and Molecular Biology
https://doi.org/10.1590/1678-4685-GMB-2025-0241

Supplementary Material to "The hologenome concept and its implications for animal breeding in the multi-omics Era"

Box 1 - Analytical challenges and integration strategies in hologenomic research.

Hologenomic research integrates multiple omic layers from the host and its associated microbiota. These data layers enable a systems-level understanding of how host-microbiome interactions shape phenotypes of interest.

Core Challenges: The integration of holo-omic data is challenged by high dimensionality, limited sample sizes, heterogeneity across data types, and the compositional nature of microbiome data. Additional constraints arise from reliance on curated knowledge bases, which differ in annotation depth and nomenclature.

Integration Strategies: Studies employ early, intermediate, and late data integration approaches. Intermediate methods are particularly valuable for modeling cross-omic dependencies. In addition, advanced statistical models, machine learning, and network-based analyses are used to reduce dimensionality and infer biologically meaningful interactions.

Network Inference: Network-based frameworks further enable the identification of key regulatory nodes, such as hubs and keystone taxa, supporting hypothesis generation regarding causal relationships within hologenomic systems.
